# An Asian case of combined 17α-hydroxylase/17,20-lyase deficiency due to homozygous p.R96Q mutation: A case report and review of the literature

**DOI:** 10.3389/fendo.2022.989447

**Published:** 2022-10-19

**Authors:** Qian Liao, Rufei Shen, Mingyu Liao, Chenxi Ran, Ling Zhou, Yuling Zhang, Guiliang Peng, Zheng Sun, Hongting Zheng, Min Long

**Affiliations:** ^1^ Department of Endocrinology, Translational Research Key Laboratory for Diabetes, The Second Affiliated Hospital, Xinqiao Hospital, Army Medical University, Chongqing, China; ^2^ Department of Medicine, Division of Diabetes, Endocrinology and Metabolism, Baylor College of Medicine, Houston, TX, United States; ^3^ Department of Molecular and Cellular Biology, Baylor College of Medicine, Houston, TX, United States

**Keywords:** congenital adrenal hyperplasia (CAH), 17α-hydroxylase/17,20-lyase deficiency, p.R96Q mutation, 46,XY disorder of sex development, inguinal hernia

## Abstract

**Background:**

Combined 17α-hydroxylase/17,20-lyase deficiency (17-OHD) is a very rare form of congenital adrenal hyperplasia (CAH) caused by mutations in the CYP17A1 gene. Almost 100 different mutations of the CYP17A1 gene have been reported, including p.R96Q mutation, but no case of p.R96Q mutation has been described in Asian populations.

**Case presentation:**

We describe a 22-year-old female patient of 46,XY karyotype, who presented with pseudohermaphrodism, primary amenorrhea, underdeveloped secondary sexual characteristics, delayed epiphyseal healing, hypertension, and hypokalemia. The diagnosis of 17-OHD was reached by measurement of steroid hormones and abdominal CT scan and confirmed by genetic sequencing, which revealed a homozygous p.R96Q missense mutation in the CYP17A1 gene. The patient received treatment with dexamethasone and estradiol, and 4 months of follow-up showed that both blood pressure and potassium were well controlled.

**Conclusions:**

This is the first Asian case of CAH caused by a homozygous p.R96Q missense mutation in the CYP17A1 gene. Herein, we highlight the role of inguinal hernia in the early diagnosis of female 17-OHD and the necessity of removing the ectopic testis.

## Introduction

Combined 17α-hydroxylase/17,20-lyase deficiency (17-OHD) is a rare autosomal recessive disease, caused by mutations in the CYP17A1 gene (1); it accounts for nearly 1% of all cases of congenital adrenal hyperplasia (CAH) (2). Steroid 17α-hydroxylase/17,20-lyase catalyzes both the 17α-hydroxylation of pregnenolone and progesterone and the 17,20-lyase reaction of 17α-hydroxypregnenolone and 17α-hydroxyprogesterone to produce the steroid precursors of androgens and estrogens. 17-OHD is associated with impaired production of cortisol and sex steroids, leading to an overproduction of plasma adrenocorticotropic hormone (ACTH) and mineralocorticoids, resulting in hypertension, hypokalemia, bilateral adrenal hyperplasia, and disorders of sexual development (DSDs) (3). Rare as it is, with the rapid improvement in diagnostic methods, more and more cases have been reported; to date, more than 100 different mutations of the CYP17A1 gene have been reported to cause complete or partial 17-OHD. In 2006, Brooke et al. described the first case of p.R96Q mutation on exon 1 of the CYP17A1 gene in an Arabian female patient, who demonstrated clinical and biochemical features of combined 17alpha-hydroxylase/17,20-lyase deficiency (4). Since then, several cases of 17-OHD due to p.R96Q mutation have been reported worldwide (5–8). Herein, we report the first Asian case of 17-OHD, caused by a p.R96Q mutation in the CYP17A1 gene.

## Case description

A 22-year-old female college student was admitted to our hospital with complaints of primary amenorrhea, fatigue, and constantly growing taller after puberty. Before junior high school (15 years old), her height was similar to that of her peers, but she showed constant growth by 2–3 cm annually between age 15 and 18 years old and 1–2 cm every year thereafter. She has a medical history of hypokalemia and hypertension for over 10 years, which were not well treated. At 3 years old, she received surgery for left inguinal hernia. She was the first child of non-consanguineous parents. The patient has a younger sister who is 12 years old and underwent menarche at 11 years old with development of breasts and axillary hair.

On physical examination, the patient was quite tall, with a height of 171 cm, weight of 59.9 kg, and BMI of 20.5 kg/m^2^. Her blood pressure was elevated at 164/122 mmHg, and her heart beat was 111 beats/min. The skin color was dark yellow, there was no growth of breasts and no axillary hair, and the external genitalia was of female type but with no pubic hair.

**Figure 1:**
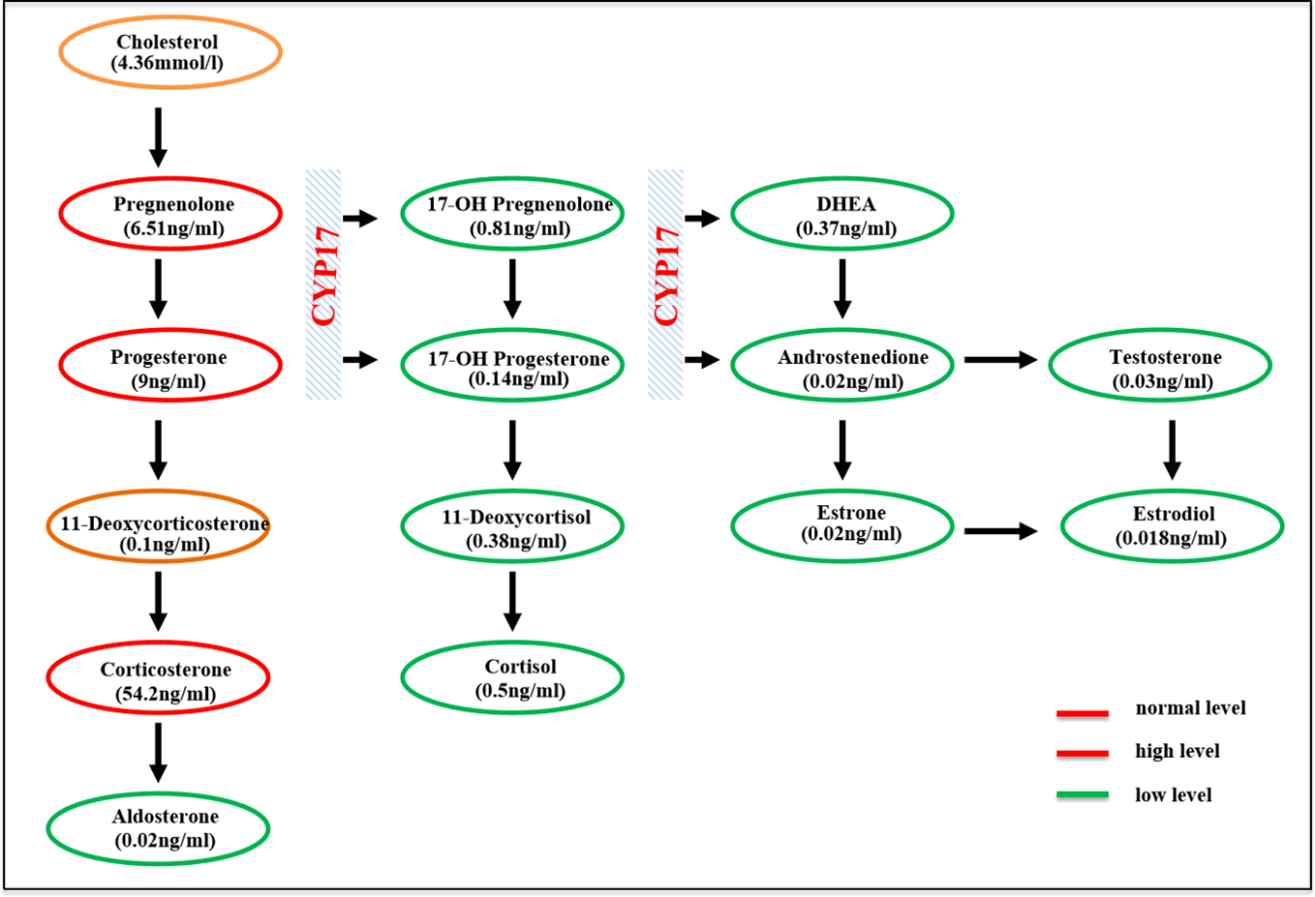
Schematic of the adrenal steroidogenesis pathway.

The clinical, biochemical, and hormonal parameters of this case were obviously abnormal. Specifically, serum potassium was decreased to 2.8 mmol/L; however, 24-h urinary potassium was increased to 51.9 mmol/24 h. Serum cortisol measurements at 08:00, 16:00, and 00:00 were all decreased below the test threshold (<27.6 nmol/L) with a high plasma ACTH level of 133 ng/L. The sex hormones dehydroepiandrosterone sulfate (DHEAS) and estradiol were low; however, serum luteinizing hormone (LH) and follicle-stimulating hormone (FSH) levels were elevated to 45.71 and 86.86 IU/L, respectively. Serum aldosterone was 42.8 pg/ml, and plasma renin activity was 0.01 ng/ml/h. The glomerular filtration function was slightly decreased (eGFR 71 ml/min/L), but liver transaminase, cholesterol, blood glucose, HbA1c, and thyroid profiles were within the normal range. Clinic manifestations and general blood examination results are summarized in **Table 1**. Steroid hormones were further measured simultaneously by liquid chromatography–tandem mass spectrometry using an ARCHITECT i2000sr (Abbott Diagnostics, Abbott Park, IL, USA) in a standardized manner. To give a clear understanding of the steroid metabolites, the steroid hormones are listed according to the steroid metabolism pathway in **Figure 1**, and the reference range of each steroid hormone is summarized in **Supplementary Table 1**. Briefly, serum cortisol, androstenedione, estradiol, and aldosterone were drastically decreased. Serum concentrations of 17-OH pregnenolone (0.81 ng/ml) and 17-OH progesterone (0.14 ng/ml) were low, but pregnenolone (6.51 ng/ml), progesterone (9 ng/ml), and corticosterone (54.2 ng/ml) were raised; 11-deoxycorticosterone was in the normal range (0.1 ng/ml).

**Table 1:** Clinical manifestations and general blood examination results in this case.

Item	Result	Reference range	Item	Result	Reference range
Age (years)	22		ACTH (pg/ml)	133	7.2–63.3
Social sex	Female		Cortisol (nmol/L)*		
Karyotype	46, XY		8:00	< 27.6	101.2–535.7
Height (cm)	172		0:00	< 27.6	
Body weight (kg)	59.9		16:00	< 27.6	79–447.8
Blood pressure (mmHg)	164/122	130/80	DHEAS (µg/dl)	41.4	74.8–410.2
Sodium (mmol/L)	148	135–145	FSH (mIU/ml)	86.86	0.95–1.95
Potassium (mmol/L)	2.8	3.5–5.5	LH (mIU/ml)	45.71	1.14–8.75
urinary potassium (mmol/24 h)	51.90	25–110	GH (µg/L)	0.66	0–8
ALT (IU/L)	25.9	7–40	IGF-1 (ng/ml)	264	116–358
AST (IU/L)	20.7	13–35	Thyroid hormone		
eGFR (ml/min/L)	71	>90	FT3 (pmol/L)	4.69	2.43–6.01
Aldosterone (pg/ml)*	42.8	70–300	FT4 (pmol/L)	14.17	9.01–19.05
Plasma renin activity (ng/ml/h)	0.01	0.1–6.56	TSH (mIU/L)	7	0.35–4.94

*Aldosterone and cortisol were measured by chemiluminescence assay.

Considering the possibility of disorders in sexual development, medical imaging examinations and karyotype analysis were carried out. Abdominal–pelvic CT scan and ultrasound revealed no visible internal genitalia, such as vagina, ovaries, uterus, or testicles but found bilateral adrenal hyperplasia; the bone age was delayed to 11 years old, and the bone mineral density values were computed using dual-energy x-ray absorptiometry images (DPX-NT, GE Healthcare); T-score of the lumbar spine (L1-L4) was −3.5, which implied osteoporosis, and T-score of the left femoral neck was −1.6, which implied osteopenia. The karyotype was 46,XY, indicative of male sex.

To gain a clear perception of this disease, informed consent was obtained from the patient. Genomic DNA was extracted from peripheral blood leukocytes using a Blood DNA mini kit (Qiagen, Germany); DNA fragments in the target region were enriched using a customized capture kit (MyGenostics, Beijing, China) and sequenced using a high-throughput second-generation sequencing platform (DNBSEQ-T7, MGI, Shenzhen, China). Sanger sequencing was used to confirm the mutations. The analysis revealed a homozygous mutation on exon 1 of the CYP17A1 gene at nucleotide 287, which was changed from guanine G to adenine A (c.287G>A), resulting in the amino acid at codon 96 changing from arginine to glutamine (p.R96Q), which caused the complete 17-hydroxylase/17,20-lyase deficiency. For personal reasons, her parents and sibling were unwilling to be tested, so we did not investigate this mutation in her relatives.

**Figure 2:**
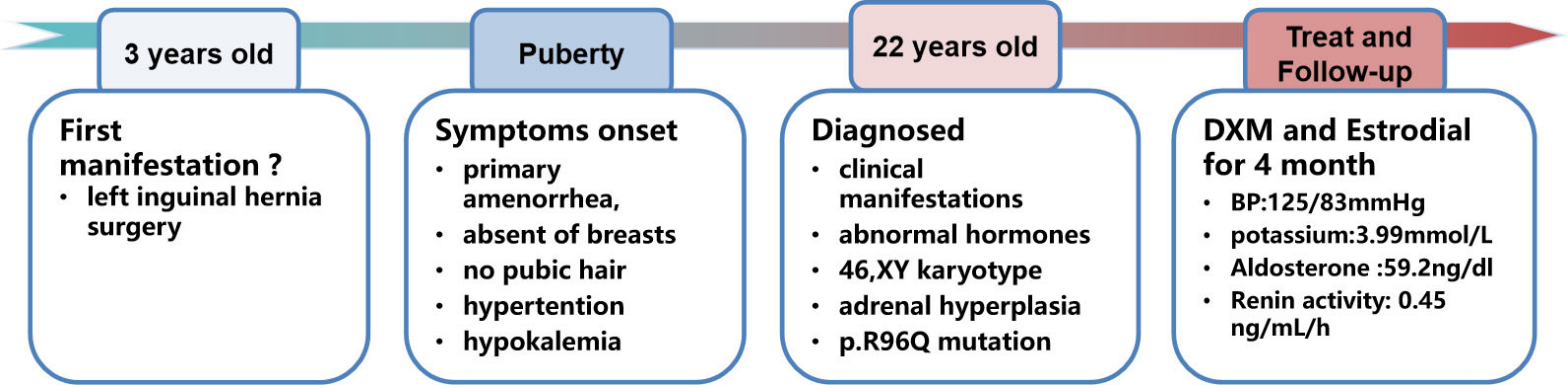
Timeline of the disease progression.

Once the diagnosis was made, oral dexamethasone at 1 mg/day was prescribed to treat the hypertension and hypokalemia. After 1 month of dexamethasone treatment, the blood pressure had decreased to 120/90 mmHg and the potassium increased to 3.29 mmol/L; the skin color was whiter, and the patient felt more energetic. With regard to the choice of gender, we fully communicated with the patient and her parents, and the patient fully understood the condition and identified herself as being of female gender. Estrogen replacement was initiated 1 month later to promote epiphyseal healing and maintain the female appearance. With regard to the uncertain existence of an ectopic testis, in case of malignant change, we recommended that the testis need to be searched for and surgically removed, but the patient refused to undergo laparoscopic exploration. On follow-up examination at 4 months, the hypertension and hypokalemia were totally resolved (Blood pressure (BP), 125/83 mmHg; K^+^, 3.99 mmol/L), and plasma renin activity was apparently elevated (0.45 ng/ml/h) with a slight elevation of aldosterone (59.2 ng/dl). We further tested the level of serum anti-Müllerian hormone (AMH), alpha-fetoprotein (AFP), and beta-human chorionic gonadotropin (beta-HCG); the result for AMH was 3.4 ng/ml (reference range, 0.96–13.34 ng/ml), beta-HCG was 3.69 mIU/ml (reference range, <5 mIU/ml), and AFP was 1.8 ng/ml (reference range, 0–10 mg/ml). The timeline of the disease progression is shown in **Figure 2**.

## Discussion

To the best of our knowledge, this is the first case in the Asian region of a homozygous p.R96Q mutation of the CYP17A1 gene, leading to combined 17-OHD. In 2006, Brooke et al. described the first case of p.R96Q mutation in the CYP17A1 gene in a 17-year-old 46,XX female patient from the United Arab Emirates (4). Nine years later, her three affected siblings were presented at a hospital with hypertension and were found to have the same missense mutation. The twin siblings were diagnosed at the age of 14 years: one of them presented with low potassium of 3.3 mmol/l, and the other with normal level (3.8 mmol/l); another sibling was 8 years old and also had low potassium of 3.3 mmol/l (8). In 2013, Athanasoulia et al. (5) described a German case of missense homozygous mutation at codon 96 (p.R96Q) of the CYP17A1 gene, causing a 46,XY DSD. Their patient presented with amenorrhea, hypertension, and normokalemia and additionally showed lack of breast development despite high doses of estradiol replacement treatment for 3 years. In 2014, Mula-Abed et al. (6) reported a misdiagnosed case in a 22-year-old Omani woman, who was initially diagnosed with hypertension at the age of 10 years and treated with anti-hypertensive drugs for 12 years, until genetic analysis revealed CYP17A1 homozygous mutation (p.R96Q), when the diagnosis of 17-OHD was confirmed. In 2017, Camtosun et al. (7) reported another misdiagnosed case in Turkey, who presented with inguinal hernia on the right side at 2.5 years old, which was found to be testicular tissue. Given the lack of hypertension and hypokalemia, the initial diagnosis was partial gonadal dysgenesis or testosterone synthesis defect. However, at 9 years old, a low normal level of potassium and a high progesterone concentration were noticed, and a correct diagnosis was made of 17-OHD, owing to p.R96Q homozygous mutation. Laflamme et al. (9) reported two French–Canadian 46,XY pseudohermaphrodism siblings (11 and 5 years old), with mutations at the same amino acid site, suffering from 17-OHD because of a homozygous missense mutation of p.R96W, caused by a C-to-T transition converting codon 96 (CGG) encoding arginine into (TGG) encoding tryptophan. These two girls showed no signs of hypertension or hypokalemia. Costenaro et al. (10) reported a 16-year-old Brazilian patient with CYP17A1 deficiency caused by a homozygous p.R96W mutation, who sought medical care for lack of pubertal signs and primary amenorrhea; additionally, hypertension and hypokalemia were revealed. Martin et al. (11) reported another Brazilian case of 17-OHD attributable to homozygous R96W mutation; the patient was a 19-year-old 46,XY woman, who presented with sexual infantilism, hypertension, and hypokalemia. In these cases, the age at hypertension onset and the degree of hypokalemia appear to vary, even in those with the same mutations. To strengthen the understanding of 17-OHD, especially this novel subtype of mutations such as ours, their relevant clinical and biochemical data are reviewed in **Table 2**.

**Table 2:** Clinical characteristics of patients with R96W or R96Q mutations.

Mutation site	c.287G>A ((p.R96Q), homozygous								p.R96W, homozygous			
Case, reference	Our case	Athanasoulia et al. (5)	Mula-Abed et al. (6)	Camtosun et al. (7)	Brooke et al. (4)	Deeb et al. (8)			Laflamme et al. (9)		Costenaro et al. (10)	Martin et al. (11)
Patient, nationality	P0 (Asian)	P1 (Caucasian)	P2 (Oman)	P3 (Turkey)	P4 (Arab)	P5 (Arab)	P6 (Arab)	P7 (Arab)	P8 (Canadian-French)	P9 (Canadian-French)	P10 (Brazilian)	P11 (Brazilian)
Diagnosis age (years)	22 (3)*	17	22	9 (2.5)*	17	14	14	8	5	11	16	19
Presenting feature	Primary amenorrhea (inguinal hernia)*	Primary amenorrhea	Hypertension	Low normal potassium (inguinal hernia)*	Germ cell tumor	Family screening	Headache hypertension	Hypertension	Inguinal hernia	Family screening	Primary amenorrhea	Sexual infantilism
Karyotype	46,XY	46,XY	46,XY	46,XY	46,XX	46,XX	46,XX	46,XX	46,XY	46,XY	46,XY	46,XY
8:00 Cortisol (nmol/L)	< 27.6	63.9	< 30	/	15	/	/	/	20	16	50	/
ACTH (pg/ml)	133	77.1	78.8	/	21	55.7	35	198	/	/	476	99
FSH/LH (IU/L)	86.86/45.71	73.7/77.7	60.5/18.3	13.99/2.19	32/60	/	/	/	85/16	63/55	46.6/60.1	34/26
Testosterone (ng/ml)	0.03	<0.03	0.01	0.928	0.12	Undetectable	Undetectable	Undetectable	/	/	< 0.1	2.6
DHEAS (µg/dl)	41.4	1.91	0.67	5.59	Undetectable	Undetectable	Undetectable	Undetectable	0.2	0.2	2	Undetectable
Estradiol (pg/ml)	0.39	5	< 9.5	11.89	88	/	/	/	/	/	< 5	< 10
17-OHP (ng/ml)	0.14	0.3	0.17	0.04	Undetectable	1.8	Undetectable	Undetectable	1.3	1.4	1.8	0.2
Potassium (mmol/L)	2.8	Normal	Normal	3.7	2.5	3.8	3.3	3.3	3.5	3.8	3.2	3.3
Blood pressure (mmHg)	164/122	120/100	145/90	Normal	170/96	163/117	155/110	153/85	Normal	Normal	150/115	150/105
Treatment	DXM, estradiol	HC, estradiol	DXM, estradiol	HC	Tumor resection, PREDNIS, estradiol	Anti-HP, HC, estradiol		HC	Not recorded		PREDNIS, estradiol	Not recorded

DXM, dexamethasone; HC, hydrocortisone; PREDNIS, prednisolone; Anti-HP, antihypertensive drugs.

*Our patient was diagnosed of 17-OHD at age 22 years, because of primary amenorrhea, but she should have been diagnosed when an inguinal hernia was detected at age 3 years. Same as the case reported by Camtosun et al.

Most cases of 17-OHD are diagnosed after adolescence because of abnormal sexual development, hypertension, and hypokalemia. Given that blood pressure and serum potassium are not measured routinely in pediatric practice, these patients are seldom recognized at prepubertal ages; furthermore, according to one report, 10%–15% of patients with 17-OHD may not have hypertension and hypokalemia for life (12). As we summarized above, although hypertension is the most common clinical sign in patients with p.R96Q or p.R96W mutations (nine of 12 cases), three of the 12 cases remained normotensive, and six of the 12 cases had normal potassium concentration at the time of diagnosis. Therefore, hypertension and hypokalemia may not be the best indicators, and another earlier distinctive indicator is required. Although no specific proportion of 17-OHD cases with inguinal hernia has been reported, there are several cases which were misdiagnosed who had a reported surgical history of inguinal hernia in childhood. Abad et al. reported a 46, XY woman who was diagnosed with 17-OHD at 23 years old because of primary amenorrhea, along with severe hypertension and hypokalemia; medical history review showed an operation for bilateral inguinal hernia at 8 years old, presumably for an undescended testis (13). Katsumata et al. reported a Japanese girl of 46,XY phenotype who received inguinal hernia surgery at the age of 4 years; biopsy showed testicular tissue, but steroid hormones and CYP17 gene mutations were not investigated until hypertension was clearly recognized 2 years later (14). Satoh et al. reported a 46,XY phenotypic girl who was diagnosed with17-OHD at the age of 6 years because of hypertension, whereas medical history showed that she had right-sided inguinal hernia at the age of 4 years (15). Likewise, Camtosun et al. reported a misdiagnosed case of 17-OHD with a mutation at p.R96Q in whom inguinal hernia on the right side was the earliest manifestation (7). Laflamme et al. (9) reported another patient with 17-OHD caused by p.R96W mutation, who was initially brought to clinical attention at the age of 5 years for left inguinal hernia; further examination showed undetectable testosterone and estradiol.

Review of the medical history of our patient showed that, when she was 3 years old, she underwent surgery for left inguinal hernia, although the patient could not provide the pathologic biopsy result of this inguinal tissue, we consider it was most likely to be immature testicular tissue. In light of these findings, early-onset inguinal hernia may be another important clue suggesting the necessity for exploration of an underlying disorder such as 17-OHD, especially in phenotypically female children. Adrenal steroid profile and chromosome karyotype analysis should be investigated early, especially in those prepubertal female children with inguinal hernia; despite the absence of hypertension or remarkable hypokalemia, genetic testing is important for confirmation.

It is well known that female patients with Y-chromosome material in their karyotypes are at high risk of gonadal tumor development, with a prevalence of approximately 18.3% (16). Given its rarity and the degree of underreporting, there are no specific data on the gonadal malignancy risk for patients with 17-OHD. In 2015, Jiang et al. reported that two of 20 (10%) 17-OHD patients with 46,XY karyotype were diagnosed with gonadal tumors: one with a Leydig cell tumor and the other with a Sertoli cell tumor (17). Another report showed that two of the 22 cases of 46,XY karyotype 17-OHD patients were diagnosed with germ cell tumors (9.09%): one a Sertoli cell tumor and the other a dysgerminoma (18). Two Chinese patients with 17-OHD with the karyotype of 46,XY who accepted laparoscopic exploration showed undeveloped testes in the inguinal region, and the pathology showed malignant changes (19). Among the cases that we summarized, one of the patients was discovered to have 17-OHD because of the presentation with germ cell tumor (4). Searching for the existence of ectopic testicular tissue in 46,XY female patients with 17-OHD is important; if ectopic testes are found, surgical resection is required to prevent malignant transformation. Because imaging examinations cannot identify the ectopic testicular tissue and laparoscopic exploration is invasive, some biochemical parameters such as AMH and inhibin B may be helpful in determining the presence of testicular tissue in patient with DSD (20). The presence of AMH in this case indicated the existence of testicular tissue; meanwhile, serum tumor markers related to germ cell tumors such as AFP and beta-HCG were negative; therefore, we recommend that the patient be closely monitored.

In terms of medication, glucocorticoid replacement therapy is the basis of all treatments, and hydrocortisone, dexamethasone, and prednisone are all appropriate. The purpose of glucocorticoid therapy is to suppress the stimulation of the adrenocortical zona by excessive ACTH, reducing the excess of mineralocorticoid, so as to control hypertension and hypokalemia (21). With the glucocorticoid replacement therapy, hypertension and hypokalemia can be gradually relieved, so antihypertensive drugs are not generally needed, but if the patients already present with prolonged uncontrollable hypertension and target organ damage, then antihypertensive treatment is needed.

Another important aspect is the choice of gender, and treatment must follow ethical principles (22). Before receiving sex hormone replacement treatment, it is recommended that patients should consult a psychologist, have knowledge of their own disease, and determine their sexual identification; organ correction surgery is also feasible to improve the quality of life.

## Conclusion

We have reported the first Asian case of 17-OHD due to a homozygous missense mutation of p.R96Q in the CYP17A1 gene in a 22-year-old 46, XY karyotype female patient. The diagnosis of this disease is usually delayed to late adolescence or even adulthood. Our hypothesis that presentation of a female infant with inguinal hernia might represent an early sign of 17-OHD should be the subject of further studies. The ectopic testes should be sought and removed to prevent possible malignant lesions in 46,XY karyotype female patients. Early diagnosis provides a chance to induce sexual development at the appropriate time and to prevent hypertension, hypokalemia, and other related complications.

## Data availability statement

The original contributions presented in the study are included in the article/**Supplementary Material**. Further inquiries can be directed to the corresponding authors.

## Ethics statement

This study was reviewed and approved by Ethics Committee of Xinqiao Hospital, Army Medical University (Third Military Medical University). The patients/participants provided their written informed consent to participate in this study.

## Author contributions

QL contributed to the study conception and literature search and drafted the manuscript. CR, LZ, YZ, and GP performed the literature search and organized the data. RS and MYL revised the manuscript. ZS reviewed the manuscript and offered advice, HZ supervised the project and critical reviewed of the manuscript, and ML supervised the project, provided funding for the project, critical reviewed of data and the manuscript. All authors contributed to the article and approved the submitted version.

## Funding

This study was supported by Chongqing Natural Science Foundation (Outstanding Youth Foundation), No. cstc2020jcyj-jqX0017 to ML.

## Conflict of interest

The authors declare that the research was conducted in the absence of any commercial or financial relationships that could be construed as a potential conflict of interest.

## Publisher’s note

All claims expressed in this article are solely those of the authors and do not necessarily represent those of their affiliated organizations, or those of the publisher, the editors and the reviewers. Any product that may be evaluated in this article, or claim that may be made by its manufacturer, is not guaranteed or endorsed by the publisher.
