# Supplementary material for: An Asian case of combined 17α-hydroxylase/17,20-lyase deficiency due to homozygous p.R96Q mutation: A case report and review of the literature
Source: Front Endocrinol (Lausanne). 2022 Oct 19;13:989447. doi: 10.3389/fendo.2022.989447 (PMC9627194; doi:10.3389/fendo.2022.989447)
Supplement: Supplementary file 1 [file Table_1.pdf]

Supplementary table 1. The reference range of each steroid hormones tested by LC-MS/MS.

Item	Result	Reference range
Pregnenolone(ng/ml)	6.51	0.07-1.88
Progesterone(ng/ml)	9	<0.2
11-Deoxycorticosterone(ng/ml)	0.1	≤0.23
Corticosterone(ng/ml)	54.2	0.6-12.93
Aldosterone(ng/ml)	0.02	0.013-0.16
17-OH Pregnenolone(ng/ml)	0.81	0.31-4.55
17-OH Progesterone(ng/ml)	0.14	<0.8
11-Deoxycortisol(ng/ml)	0.38	<1.07
Cortisol(ng/ml)	0.5	60-260
DHEA(ng/ml)	0.37	1.02-11.85
Androstenedione(ng/ml)	0.02	0.35-2.5
Estrone(ng/ml)	0.02	0.01-0.138
Testosterone(ng/ml)	0.03	0.02-0.45
Estradiol(ng/ml)	0.018	0.013-0.166
